# The role of PEEP for cannulation of the subclavian vein: A prospective observational study

**DOI:** 10.1371/journal.pone.0285110

**Published:** 2023-04-27

**Authors:** Christine Eimer, Knut G. Rump, Gunnar Elke, Tobias H. Becher, Norbert Weiler, Amke Caliebe, Dirk Schädler

**Affiliations:** 1 Department of Anesthesiology and Intensive Care Medicine, University Medical Center Schleswig-Holstein, Campus Kiel, Kiel, Germany; 2 Institute for Medical Informatics and Statistics, University Medical Center Schleswig-Holstein, Campus Kiel, Kiel, Germany; Universitair Kinderziekenhuis Koningin Fabiola: Hopital Universitaire des Enfants Reine Fabiola, BELGIUM

## Abstract

**Purpose:**

The role of positive endexpiratory pressure (PEEP) for successful cannulation of the subclavian vein (SCV) remains inconclusive. The aim of our study was to assess the effect of different levels of PEEP on distance from SCV to parietal pleura (DVP) and on the cross-sectional area (CSA) of the SCV.

**Methods:**

Invasive mechanically ventilated adult patients with a clinical indication for a stepwise PEEP-trial (0, 5, 10, and 15 cm H_2_O) were included in this prospective observational single-center study. Ultrasound examinations of SCV were performed with a linear ultrasound probe using the infraclavicular view. DVP and CSA were measured on the right and left bodyside. Examinations were repeated at each PEEP step.

**Results:**

27 patients were enrolled (12 female; 60±21 years; BMI 24.6±4.9 kg/m^2^; 20 patients on controlled, 7 on assisted ventilation). A statistically significant increase of DVP in the in-plane view was found on the left side which was not clinically relevant. No significant differences of DVP were observed in all other views. PEEP induced changes in CSAs were statistically significant but clinically not relevant on both sides. The largest change in CSA (2mm^2^) was observed when comparing PEEP 10 with PEEP 0 cm H_2_O.

**Conclusion:**

A stepwise PEEP increase was not associated with clinically relevant changes of the DVP and CSA. Thus, a PEEP-optimization for the cannulation of the subclavian vein is not indicated.

## Introduction

The role of positive endexpiratory pressure (PEEP) for successful cannulation of the subclavian vein (SCV) remains inconclusive [1, 2]. A higher PEEP may decrease distance from SCV to the parietal pleura consecutively increasing the periprocedural risk of pneumothorax. In contrast, a higher PEEP could also lead to a higher cross-sectional area (CSA) of SCV which may increase the punction success rate. The aim of this study was to assess the effect of different levels of PEEP on distance from SCV to parietal pleura (DVP) and on CSA.

## Methods

This prospective observational single-center study was conducted in three surgical intensive care units of the University Medical Center Schleswig-Holstein, Campus Kiel, Germany. The study was approved by the ethics committee of the Christian-Albrechts University Kiel, Germany (approval D568/19) and registered (www.drks.de; DRKS00023173). Need for informed consent was waived by the ethics committee. Study data were analyzed anonymously. Recruitment lasted from March 2020 until February 2021. Invasive mechanically ventilated adult patients with a clinical indication for a stepwise PEEP-trial (0, 5, 10, and 15 cm H_2_O) were eligible. Exclusion criteria were already inserted lines in at least one SCV or PEEP>15 cm H_2_O.

Ultrasound examinations were performed by an experienced investigator with a linear ultrasound probe (9-L-D; 2,4 MHz, General Electric Healthcare, Munich, Germany) using the infraclavicular view. Patients were placed in the 30° semi recumbent position with head turned slightly to the contralateral side. Sonographic imaging was used to determine where the subclavian vein appears below the clavicle. The necessary measurements were taken at this location. SCV was scanned in plane and out-of-plane. For the in-plane image, the transducer was rotated in this location so that the clavicle was still visible on the image. DVP and CSA were measured on the right and left bodyside. The largest CSA of several respiratory cycles was selected. Examinations were repeated at each PEEP step. When PEEP was changed, ultrasound measurements were performed with a time delay of 2 minutes to ensure effectiveness of the PEEP change.

### Statistical considerations

For sample size calculation, comparison of DVP at PEEP 0 cm H_2_O and PEEP 15 cm H_2_O was considered as the primary objective. A change in DVP > 2 mm was considered clinically relevant and defined as the primary study endpoint. Normal distribution of the paired differences of distance was assumed. According to previously published data [3], a standard deviation for the paired differences of 3 mm was selected. For a significance level of 0.05 and a power of 0.90, the required sample size was calculated to be 26 (BIAS for windows version 11.12). The software R V4.0.3 [4] and R package nlme [5] were used for all analyses. All tests were performed two-sided at a significance level of 0.05. A change in CSA > 2 mm^2^ was considered clinically relevant. For the comparison of DVP and CSA at PEEP 0, PEEP 5, PEEP 10 and PEEP 15 cm H_2_O, a paired t test was applied. For the influence of all PEEP steps on the DVP and CSA, a linear mixed model approach was chosen with PEEP modelled as fixed effect and individuals as random effect. Based on significance, either both random slope and intercept or only random intercept were included in the models. For all mixed models the random effect was significant (p<0.001). The covariables gender, age, body mass index (BMI) and ventilation mode were assigned fixed effects. The mixed model was performed for each influence variable separately and in a multiple fashion. As only one variable remained in the multiple model after backward selection (p < 0.05), only the results of the univariable analyses for the influence variables are considered.

## Results

**Fig 1:**
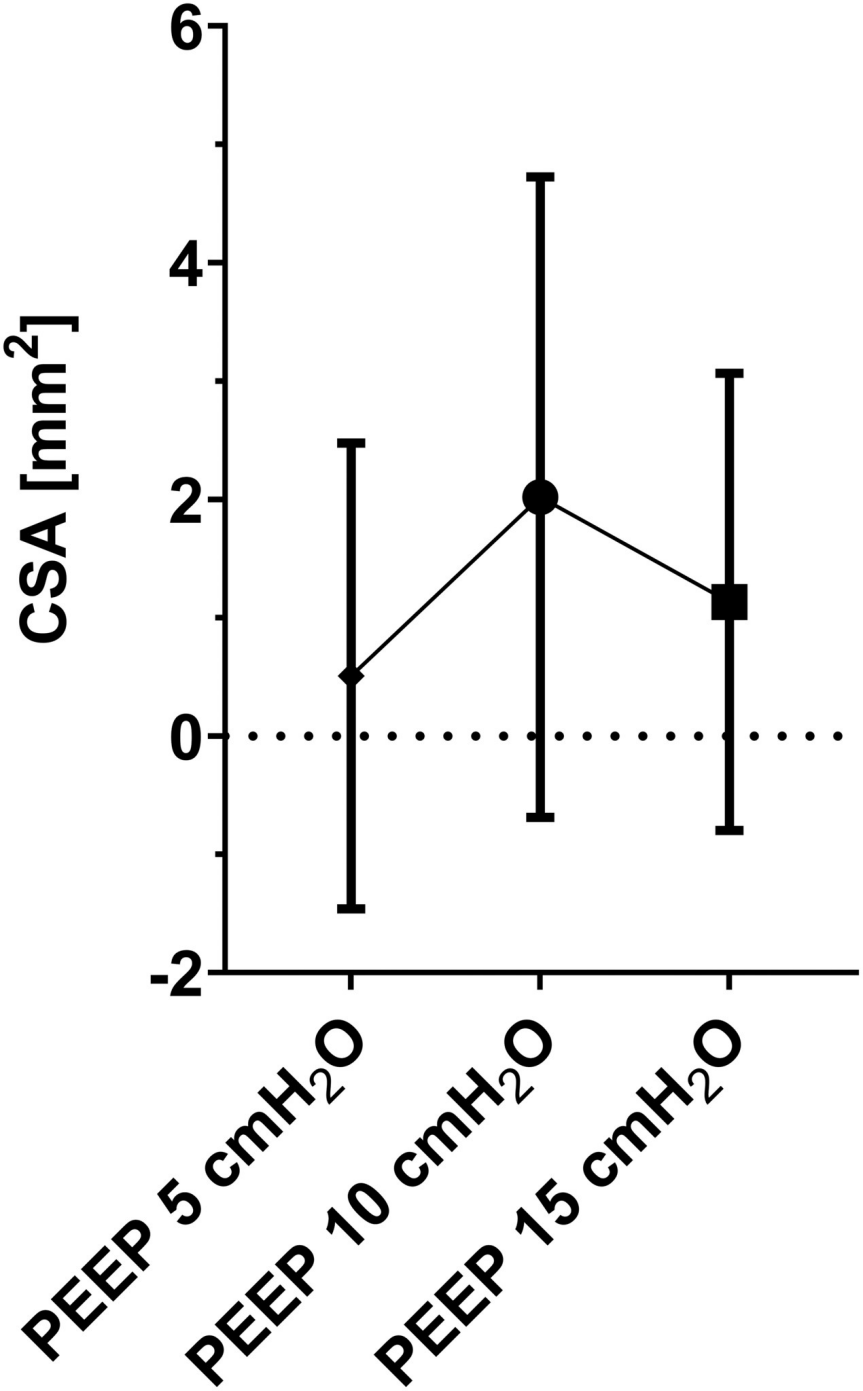
Change of the subclavian vein cross-sectional area (CSA) measured during different positive endexpiratory pressure (PEEP) steps. At each PEEP step, the measured CSA at PEEP 0 cm H_2_O was subtracted from the measured CSA value at the PEEP step. All values were pooled for the left and the right body side and are shown as means and standard deviations.

27 patients were enrolled (12 female; 60±21 years; BMI 24.6±4.9 kg/m^2^; 20 patients on controlled, 7 on assisted ventilation). Measured values for DVP and CSAs are given in Table 1. For our primary objective (the difference between PEEP 0 and PEEP 15) we found a statistically significant increase of DVP in the in-plane view on the left side that was not clinically relevant. No significant differences were observed in all other views. PEEP induced changes in CSAs were statistically significant on both sides. The largest change in CSA (2mm^2^) was observed when comparing PEEP 10 with PEEP 0 cm H_2_O (Fig 1). P values for pairwise comparisons of PEEP levels for distance between subclavian vein and parietal pleura and cross-sectional area of the subclavian vein are summarized in S1 Table. The mixed model revealed that PEEP influenced DVP in plane left (p = 0.036), CSA right (p = 0.007) and CSA left (p = 0.001) significantly. Age was associated with right DVP out of plane (p = 0.049), BMI with left DVP out of plane (p = 0.014). Gender and ventilation mode showed no significant effects.

**Table 1:** Distance between subclavian vein and parietal pleura and cross-sectional area of the subclavian vein measured during different PEEP settings.

Variable	PEEP 0 cm H_2_O	PEEP 5 cm H_2_O	PEEP 10 cm H_2_O	PEEP 15 cm H_2_O	PEEP 15—PEEP 0 cm H_2_O	P Value*
DVP in plane right [mm]	8.8±3.4	9.4±3.1	9.2±3.3	9.4±3.7	0.6±1.6	0.055
DVP in plane left [mm]	8.5±2.8	8.6±2.4	8.9±2.4	9.2±2.5	0.7±1.8	**0.048**
DVP out of plane right [mm]	8.2±2.6	8.6±2.8	8.1±2.7	8.3±2.5	0.1±2.0	0.786
DVP out of plane left [mm]	8.0±3.8	8.2±2.7	7.7±2.3	7.8±1.9	-0.1±2.6	0.772
CSA out of plane right [mm^2^]	5.6±2.5	6.2±2.4	8.1±2.7	6.8±2.3	1.3±2.4	**0.01**
CSA out of plane left [mm^2^]	5.6±2.5	6.0±2.40	7.1±3.2	6.6±2.8	1.0±1.4	**0.001**

**Definition of abbreviations:** Cross sectional area of the subclavian vein, CSA; distance between subclavian vein and pleura parietalis, DVP; positive endexpiratory pressure, PEEP.

* Comparison of PEEP 0 cm H_2_O and PEEP 15 cm H_2_0

## Discussion

This study revealed that a higher PEEP was significantly associated with a larger distance between SCV and PP in the in-plane view on the left body side. However, the observed changes were small and not clinically relevant. Until now, no data exist regarding the effect of a PEEP-step maneuver on DVP in critically ill patients.

Ultrasound is widely used in the critical care setting for various purposes focusing on organ examination, vascular access and regional anesthesia. Regarding central venous access, current European and North-American guidelines recommend ultrasound-guided catheterization of central veins [6, 7]. The PERSEUS guidelines, initiated by the European Society of Anesthesiology provide recommendations regarding safety and effectiveness of ultrasound guided vascular access [7]. Following the literature, it becomes obvious, that ultrasound visualization of the vessel raises first-attempt success, reduces the number of complications dramatically and has therefore been proposed as routine practice for cannulation [8]. A recent study reported that ultrasound-guided cannulation can easily be implemented in clinical routine [9]. This may also be due to an increasingly integration of ultrasound education during medical school in nearly all medical disciplines [10–12], which enables young physicians to learn ultrasound-guided techniques more easily.

Central venous lines are primarily placed in the internal jugular vein, subclavian vein or femoral vein. The catheterization of the subclavian vein is associated with a higher risk of pneumothorax and a higher risk of insertion failure compared to alternative catheterisation sites [13]. Ultrasound enables to visualize anatomy of the vessels and surrounding tissue very precisely and may also give insights in topographic changes due to mechanical ventilation. However, the influence of mechanical ventilation on DVP and CSA of the subclavian vein has not yet been systematically studied. Kwon and coworkers showed that increasing PEEP from 0 to 20 cm H_2_O decreased DVP by 1 mm in 30 elective cardiac surgery patients [14]. This is in contrast to our findings where DVP increased with higher PEEP. Differences may be attributed to the varying PEEP steps used and the different patient populations studied, however, the described changes in DVP were not clinically relevant. Increasing PEEP from 0 to 10 cm H_2_O was associated with an increase of CSA while a further PEEP increase from 10 to 15 cm H_2_O CSA was not, although still significantly higher as opposed to PEEP 0 cm H_2_O. This U-shape coherence is likely explained by the higher intrathoracic pressure at a certain PEEP cut-off value leading to SCV compression. Kwon and coworkers reported a significantly higher and clinically relevant change in CSA in their cohort between PEEP 0 cm H_2_O in supine position and PEEP 20 cm H_2_O in Trendelenburg posture. A final explanation for the U-shape cannot be drawn from our results as we did not measure right-atrial and intrapleural pressures as well as the pressure in the subclavian vein. PEEP steps were always changed in the same order. Changing PEEP randomly may also have an influence and should be further investigated.

Regarding the measured values for DVP, our results are comparable with a large sonoanatomy study in 150 cardiac surgery patients [15]. Lavallée et al. report in their study mean DVP values ranging from 7 to 9 mm. In this study, mean DVP values ranged from 7.7 to 9.4 mm. However, applied PEEP levels and CSA values were not reported in the study by Lavalée [15]. The small differences in the range of a few millimeters between posterior wall of the vena subclavia and pleura parietalis underline the recommendation that visualization of the needle tip appears to be highly advisable and useful. Furthermore, it seems reasonable to follow a protocol-based algorithm: Brescia et. al have published a seven-step strategy to minimize periprocedural complications and propose a preliminary ultrasound assessment of the puncture site to evaluate anatomy, which is also in line with the ESA recommendations [7, 16]. In this regard, sonographic imaging of the subclavian vein before puncture may also be required to determine vessel collapsibility during respiratory cycle and regarding volume status [17], as a certain collapsibility of the vessel may also complicate venipuncture and increase the risk for adverse events. Periprocedural ultrasound may also serve for verification of insertion failure or life-threatening complications such as the pneumothorax, following several studies and meta-analysis, ultrasound of lung and pleura is superior to chest radiography regarding the detection of pneumothorax [18, 19]. In this sense, ultrasound examination techniques may also be used to verify the correct intravenous localization and direction of the guidewire before dilatation and may even be used to predict the correct length of the catheter in the lower third of the superior vena cava [20].

In this study, the rate of successful cannulation of SVC was not determined. However, as no clinically relevant changes in DVP and CSA were detected, a PEEP optimization for cannulation of SVC does not seem to translate into a higher rate of successful cannulations of SVC. Intraobserver and interobserver variability could not be determined in this study as the measurements were performed only once by one examiner. However, the location of the measurement was precisely defined. At the early beginning of the study, the ultrasound studies were analyzed by the study team and no relevant deviations regarding DVP, CSA were found. The axillary vein may often be misrepresented as the SCV in clinical practice when using ultrasound to access the vessel in the infraclavicular regions [21]. Although the inflow of the cephalic vein was not identified, the measurement was performed as far proximal as possible in order to actually measure SCV. Several studies reported, that venipuncture from the supraclavicular approach might be associated with less adverse events compared to the infraclavicular approach [22, 23]. This topic should be investigated in future studies especially regarding patients with mechanical venilation.

## Conclusion

Ultrasound enables to visualize anatomy of the vessels and surrounding tissue very precisely and may also give insights in topographic changes due to mechanical ventilation. In this study, sonographic imaging was used to visualize the effects of PEEP changes on the extrathoracic vascular system. A stepwise PEEP increase was not associated with clinically relevant changes of the DVP and CSA. Thus, a PEEP-optimization for the cannulation of the subclavian vein does not seem to be indicated. Further studies may investigate clinical safety and efficiency of ultrasound guided infraclavicular vs. supraclavicular SVC cannulation compared to blind cannulation.

## Supporting information

S1 Data Minimal anonymized data set. (XLSX)

S1 Table P values for pairwise comparisons of PEEP levels for distance between subclavian vein and parietal pleura and cross-sectional area of the subclavian vein measured during different PEEP settings. (DOCX)

S1 Checklist STROBE statement—Checklist of items that should be included in reports of observational studies. (DOCX)

S1 File Study protocol English. (PDF)

S2 File Study protocol German. (PDF)
