# Supplementary material for: The role of PEEP for cannulation of the subclavian vein: A prospective observational study
Source: PLoS One. 2023 Apr 27;18(4):e0285110. doi: 10.1371/journal.pone.0285110 (PMC10138469; doi:10.1371/journal.pone.0285110)
Supplement: S1 Table — (DOCX) [file pone.0285110.s002.docx]

| **Table S1.** P values for pairwise comparisons of PEEP levels for distance between subclavian vein and parietal pleura and cross-sectional area of the subclavian vein measured during different PEEP settings. | | | | | | |
| --- | --- | --- | --- | --- | --- | --- |
| Variable | PEEP 0/ PEEP 5 | PEEP 0/ PEEP 10 | PEEP 0/ PEEP 15 | PEEP 5/ PEEP10 | PEEP 5/  PEEP 15 | PEEP 10/ PEEP 15 |
| DVP in plane right | **0.044** | 0.124 | 0.055 | 0.489 | 0.934 | 0.519 |
| DVP in plane left | 0.686 | 0.251 | **0.048** | 0.521 | 0.180 | 0.318 |
| DVP out of plane right | 0.137 | 0.877 | 0.786 | 0.205 | 0.481 | 0.615 |
| DVP out of plane left | 0.559 | 0.697 | 0.772 | 0.052 | 0.202 | 0.822 |
| CSA out of plane right | 0.154 | **0.042** | **0.010** | 0.617 | 0.083 | 0.051 |
| CSA out of plane left | 0.250 | **<0.001** | **0.001** | **0.007** | 0.135 | 0.100 |
| **Definition of abbreviations:** Cross sectional area of the subclavian vein, CSA; distance between subclavian vein and pleura parietalis, DVP; positive endexpiratory pressure, PEEP. | | | | | | |
| P values from paired t-tests | | | | | | |
