# Supplementary material for: The role of PEEP for cannulation of the subclavian vein: A prospective observational study
Source: PLoS One. 2023 Apr 27;18(4):e0285110. doi: 10.1371/journal.pone.0285110 (PMC10138469; doi:10.1371/journal.pone.0285110)
Supplement: S1 File — (PDF) [file pone.0285110.s004.pdf]

# SUBCLAVIAN STUDY

Study protocol

**Objective of the study:**
Ultrasound guided placement of a central venous catheter into the subclavian vein is part of the daily routine in anesthesia and intensive care medicine.

For many years it has been assumed that adjustment of positive end-expiratory pressure (PEEP) changes the position of the subclavian vein in relation to the pleura leading to a higher risk for the occurrence of an iatrogenic pneumothorax. This has led to many subclavian vein punctures being routinely performed at a PEEP of 0 mbar. However, there is evidence that the location of the subclavian vein may be independent of the PEEP setting.

Furthermore, the subclavian vein is described as basically constant in size due to its anatomical position. This is said to be independent of the volume status of the patients. Accordingly, a change in PEEP would not influence the vascular diameter of the subclavian vein. In the meantime, however, several studies suggest that the vascular diameter changes due to respiration and fluid balance.

In this planned observational study, the position of the subclavian vein and the vessel diameter bilaterally in inspiration and expiration at different PEEP values will be examined with ultrasound. The aim is to find out to what extent PEEP reduces the distance between the subclavian vein and the pleura and whether this results in a higher risk of accidental pneumothorax. In addition, the effect of PEEP on the vascular diameter of the subclavian vein will be investigated. To draw conclusions about the patient's volume balance, the inferior vena cava will also be measured with ultrasound.

**State of research:**
There have been few studies on this topic, but a systematic evaluation at different PEEP levels is currently still pending. Previous studies in children have shown that opening the tube to atmosphere did not change the anatomical position between the subclavian vein and the pleura and thus did not increase the risk of pneumothorax (Jang et al., 2013). Furthermore, even with complete expiration, no change in the distance between the subclavian vein and pleura could be determined, but the vessel diameter changed to larger diameters (Lim et al., 2013). In a study including more than 1.000 patients, Palmaers et al. were able to show that mechanical ventilation during puncture of the subclavian vein carries a higher overall risk of pneumothorax than puncture in apnea (Palmaers et al., 2019). Another study showed that the influence of breathing on subclavian vascular diameter can be used as a predictor of volume response (Giraud et al., 2018).

**Characterisation of study participants:**
The study will enroll 60 study participants and will be conducted in the surgical intensive care units of the University Medical Center Schleswig-Holstein, Campus Kiel. The minimum age for study participation is 18 years. Patients must be ventilated via an endotracheal tube or a tracheal cannula. The change of PEEP should not lead to a worsening of the

hemodynamic or the pulmonary function leading to a change of the medical therapy. Informed consent by patients or their legal representatives is not necessary.

**Study type:**
This is a monocentric, observational study in controlled ventilated patients in the interdisciplinary surgical intensive care unit of the UKSH Campus Kiel.

**Conduct of the study:**
For the study, the study participants will be placed in semi-recumbent position. Under the current ventilation settings, the subclavian vein at the junction of the axillary vein and the subclavian vein at the level of the first rib will be displayed with the linear transducer of the ultrasound device and in longitudinal and cross-section. The diameter and the distance to the pleura will also be measured and documented. This measurement point corresponds to the puncture site for the ultrasound-guided puncture of the subclavian vein. The same procedure will be performed on the opposite side. Finally, the diameter of the inferior vena cava will be measured.

Subsequently, the PEEP level will be gradually adjusted in a target range of 0-15 mbar, whereby the difference between inspiratory pressure and PEEP should always be kept constant. Any change in PEEP will be followed by ultrasound assessments as described above. A vascular puncture is not required for the study.

The ultrasound measurements will be stopped immediately in case of hemodynamic or pulmonary instability. Hemodynamics will be monitored via continuous electrocardiography and arterial blood pressure measurement. The pulmonary function will be monitored peripheral oxygen saturation and arterial blood gas analyses. No sedative medication is required to conduct the study.

The patient data will be collected anonymously. The data will be stored in encoded form by assigning a sequential number. Data from the ultrasound examination, ventilation parameters, values from haemodynamic monitoring, the dosages of vasoactive drugs and previous illnesses will be recorded.

The collection of data is planned for the period 10/2019 to 03/2020.

References:

Giraud, R., Abraham, P. S., Brindel, P., Siegenthaler, N. and Bendjelid, K. (2018) ‘Respiratory changes in subclavian vein diameters predicts fluid responsiveness in intensive care patients: a pilot study’, *Journal of clinical monitoring and computing*, vol. 32, no. 6, pp. 1049–1055.

Jang, Y.-E., Lee, J.-H., Park, Y.-H., Byon, H.-J., Kim, H.-S., Kim, C.-S. and Kim, J.-T. (2013) ‘The effect of lung deflation on the position of the pleura during subclavian vein cannulation in infants receiving mechanical ventilation: an ultrasound study’, *Anaesthesia*, vol. 68, no. 10, pp. 1066–1070.

Lim, K.-J., Lee, J.-M., Byon, H.-J., Kim, H.-S., Kim, C.-S., Lee, S.-K. and Kim, J.-T. (2013) 'The effect of full expiration on the position and size of the subclavian vein in spontaneously breathing adults', *Anesthesia and analgesia*, vol. 117, no. 1, pp. 109–113.

Palmaers, T., Frank, P., Eismann, H., Sieg, L., Leffler, A., Schmitt, H. and Scholler, A. (2019) 'Vena-subclavia-Katheter und Pneumothoraxrisiko : Maschinelle Beatmung erhöht das Pneumothoraxrisiko während infraklavikulärer landmarkengestützter V.-subclavia-Punktion: eine prospektive randomisierte Studie', *Der Anaesthesist*, vol. 68, no. 5, pp. 309–316.
