# Supplementary material for: The role of PEEP for cannulation of the subclavian vein: A prospective observational study
Source: PLoS One. 2023 Apr 27;18(4):e0285110. doi: 10.1371/journal.pone.0285110 (PMC10138469; doi:10.1371/journal.pone.0285110)
Supplement: S2 File — (PDF) [file pone.0285110.s005.pdf]

SUBCLAVIA STUDIE

Studienprotokoll

Zielsetzung der Studie:
Die sonographisch gesteuerte Anlage eines zentralen Venenkatheters in die Vv. subclaviae gehört zur täglichen Routine in der anästhesiologischen und intensivmedizinischen Versorgung.

Seit vielen Jahren wird propagiert, dass die Einstellung des PEEP (positive end-exspiratory pressure) die Lage der V. subclavia zur Pleura verändere und somit – bei einem hohen PEEP - ein höheres Risiko für einen iatrogenen Pneumothorax bestehe. Dies hat dazu geführt, dass in der Routine viele Punktionen der V. subclavia bei einem PEEP von 0 mbar durchgeführt werden. Mittlerweile gibt es jedoch Hinweise darauf, dass die Lage der V. subclavia von der PEEP-Einstellung unabhängig sein könnte.

Darüber hinaus wird die V. subclavia durch ihre anatomische Lage als grundsätzlich größenkonstant beschrieben, dies sei unabhängig vom Volumenstatus der Patienten. Eine Veränderung des PEEP würde den Gefäßdiameter der V. subclavia dementsprechend auch nicht beeinflussen. Inzwischen legen aber mehrere Studien den Verdacht nahe, dass sich der Gefäßdiameter durch die Atmung und den Flüssigkeitshaushalt verändert.

In der geplanten Observationsstudie wird mittels Sonographie die Lage der Vv. subclaviae und der Gefäßdurchmesser bilateral in Inspiration und Exspiration bei unterschiedlichen PEEP-Werten untersucht. Es soll herausgefunden werden, inwieweit der PEEP die Distanz zwischen V. subclavia und Pleura verringert und ob dadurch ein höheres Risiko für einen akzidentiellen Pneumothorax besteht. Außerdem soll die Auswirkung des PEEPs auf den Gefäßdiameter der V. subclavia untersucht werden. Um Rückschlüsse auf den Volumenhaushalt des Patienten ziehen zu können, wird die V. cava inferior sonographisch vermessen.

Stand der Forschung:
Zu diesem Themengebiet hat es bereits vereinzelt Studien gegeben, eine systematische Erfassung bei unterschiedlichen PEEP-Werten gibt es bisher nicht.
In vorangegangenen Studien an Kindern konnte gezeigt werden, dass das Öffnen des Tubus zur Atmosphäre keine Veränderung der anatomischen Lagebeziehungen zwischen V. subclavia und Pleura bewirkt hat und somit kein höheres Pneumothoraxrisiko bestand (Jang et al., 2013). Ferner konnte auch bei kompletter Exspiration keine Veränderung der Distanz zwischen V. subclavia und Pleura ermittelt werden, es änderte sich allerdings der Gefäßdurchmesser zu größeren Diametern (Lim et al., 2013). Palmaers et al. konnten in einer Studie an über 1000 Patienten zeigen, dass eine mechanische Beatmung bei der Punktion der V. subclavia insgesamt ein höheres Risiko für einen Pneumothorax birgt, als eine Punktion in Apnoe (Palmaers et al., 2019). In einer weiteren Studie konnte gezeigt werden, dass der Einfluss der Atmung auf den Gefäßdurchmesser der V. subclavia als Prädiktor für das Ansprechen auf Volumen genutzt werden kann (Giraud et al., 2018).

Charakterisierung der Studienteilnehmer/ -innen:
In die Studie sollen 60 Studienteilnehmer/ -innen eingeschlossen werden. Die Studie wird auf den hausinternen Intensivstationen des UKSH Campus Kiel (C.110, C.111, C.112, NC4) und im operativen Bereich an invasiv beatmeten Patienten durchgeführt. Das Mindestalter für die Studienteilnahme beträgt 18 Jahre. Die Patienten müssen über einen Endotrachealtubus oder eine Trachealkanüle beatmet sein. Eine Veränderung des PEEP sollte sowohl hämodynamisch als auch pulmonal nicht zu einer therapeutisch relevanten Veränderung, einer akuten Gefährdung des Patienten oder gar zu einer Änderung des Therapieziels führen. Eine Einwilligung durch die Patienten oder deren rechtliche Vertreter ist nicht notwendig.

Studientyp:
Es handelt sich um eine monozentrische Observationsstudie bei kontrolliert beatmeten Patienten im interdisziplinären operativen Intensivbereich des UKSH Campus Kiel.

Durchführung der Studie:
Für die Studie werden die Studienteilnehmer /-innen auf dem Rücken gelagert. Unter den aktuellen Beatmungseinstellungen wird mit dem Linearschallkopf des Ultraschallgeräts die V. subclavia am Übergang der V. axillaris in die V. subclavia auf Höhe der 1. Rippe aufgesucht und in Längs- und Querschnitt dargestellt und sowohl der Diameter als auch der Abstand zur Pleura vermessen und dokumentiert. Dieser Messpunkt entspricht dem Punktionsort bei der sonographisch gesteuerten Venenpunktion der V. subclavia. Das gleiche Vorgehen erfolgt auf der Gegenseite. Darauf folgt die sonographische Darstellung des Diameters der V. cava inferior.

Im Anschluss wird der PEEP stufenweise in einem Zielbereich von 0-15 mbar eingestellt, wobei die Differenz zwischen Beatmungsdruck und PEEP immer identisch bleiben sollte. Bei jeder PEEP-Veränderung erfolgt eine Sonographie nach dem oben beschriebenen Muster. Eine tatsächliche Gefäßpunktion ist innerhalb dieser Studie nicht erforderlich.

Sollte während der Untersuchung eine Gefährdung des Patienten oder ein akutes Problem mit der Beatmung oder der Hämodynamik auftreten, wird die Untersuchung abgebrochen. Die Kontrolle der Hämodynamik erfolgt über eine kontinuierliche EKG-Ableitung, arterielle Blutdruckmessung und Überwachung der pulmonalen Funktion über Sauerstoffsättigung und arterielle Blutgasanalysen. Für die Durchführung der Studie sind keine sedierenden Maßnahmen erforderlich.

Die Patientendaten werden anonymisiert erhoben. Die Speicherung der Daten erfolgt verschlüsselt über die Vergabe einer fortlaufenden Nummer. Erhoben werden Daten aus der Ultraschalluntersuchung, Beatmungsparameter, Werte aus dem hämodynamischen Monitoring, die Dosierungen vasoaktiver Medikamente und Vorerkrankungen.

Die Erhebung der Daten ist für den Zeitraum 10/2019 bis 03/2020 vorgesehen.

Literaturüberblick:

Giraud, R., Abraham, P. S., Brindel, P., Siegenthaler, N. and Bendjelid, K. (2018) 'Respiratory changes in subclavian vein diameters predicts fluid responsiveness in intensive care patients: a pilot study', *Journal of clinical monitoring and computing*, vol. 32, no. 6, pp. 1049–1055.

Jang, Y.-E., Lee, J.-H., Park, Y.-H., Byon, H.-J., Kim, H.-S., Kim, C.-S. and Kim, J.-T. (2013) 'The effect of lung deflation on the position of the pleura during subclavian vein cannulation in infants receiving mechanical ventilation: an ultrasound study', *Anaesthesia*, vol. 68, no. 10, pp. 1066–1070.

Lim, K.-J., Lee, J.-M., Byon, H.-J., Kim, H.-S., Kim, C.-S., Lee, S.-K. and Kim, J.-T. (2013) 'The effect of full expiration on the position and size of the subclavian vein in spontaneously breathing adults', *Anesthesia and analgesia*, vol. 117, no. 1, pp. 109–113.

Palmaers, T., Frank, P., Eismann, H., Sieg, L., Leffler, A., Schmitt, H. and Scholler, A. (2019) 'Vena-subclavia-Katheter und Pneumothoraxrisiko : Maschinelle Beatmung erhöht das Pneumothoraxrisiko während infraklavikulärer landmarkengestützter V.-subclavia-Punktion: eine prospektive randomisierte Studie', *Der Anaesthesist*, vol. 68, no. 5, pp. 309–316.
